# Rewiring of neuronal networks during synaptic silencing

**DOI:** 10.1038/s41598-017-11729-5

**Published:** 2017-09-15

**Authors:** Jana Katharina Wrosch, Vicky von Einem, Katharina Breininger, Marc Dahlmanns, Andreas Maier, Johannes Kornhuber, Teja Wolfgang Groemer

**Affiliations:** 1 Department of Psychiatry and Psychotherapy, Friedrich-Alexander University of Erlangen-Nuremberg, 91054 Erlangen, Germany; 2 Pattern Recognition Lab, Department of Computer Science, Friedrich-Alexander University of Erlangen-Nürnberg, 91058 Erlangen, Germany

## Abstract

Analyzing the connectivity of neuronal networks, based on functional brain imaging data, has yielded new insight into brain circuitry, bringing functional and effective networks into the focus of interest for understanding complex neurological and psychiatric disorders. However, the analysis of network changes, based on the activity of individual neurons, is hindered by the lack of suitable meaningful and reproducible methodologies. Here, we used calcium imaging, statistical spike time analysis and a powerful classification model to reconstruct effective networks of primary rat hippocampal neurons *in vitro*. This method enables the calculation of network parameters, such as propagation probability, path length, and clustering behavior through the measurement of synaptic activity at the single-cell level, thus providing a fuller understanding of how changes at single synapses translate to an entire population of neurons. We demonstrate that our methodology can detect the known effects of drug-induced neuronal inactivity and can be used to investigate the extensive rewiring processes affecting population-wide connectivity patterns after periods of induced neuronal inactivity.

## Introduction

The effects of drug-induced silencing of neuronal activity on the morphology and activity of single synapses have been studied for decades using the voltage-gated sodium channel blocker tetrodotoxin (TTX)^1–4^. One of the most prominent findings: a strong increase in synaptic excitability^1–4^. Understanding the consequences of neuronal inactivity has furthered our understanding of synaptic architecture and function over the years and permitted the discovery of activity-independent synaptic processes. Despite Hebbian rules for the use-dependent maintenance of synapses, prolonged inactivity during TTX-treatment induces the expression of additional postsynaptic glutamate receptors^5,6^ and the enlargement postsynaptic densities^7^. Presynaptically, TTX-induced synaptic inactivity induces growth of so-called active zones, characterized by larger numbers of docked vesicles, increased spontaneous release rates, and increases in the number of vesicles released upon a stimulus^7^. Such alterations have been observed in the pathogenesis of tardive dyskinesia^8^, glaucoma^9^, neuropathic pain^10,11^, and drug addiction^12–14^ and withdrawal^15,16^. However, it remains unclear whether and how modifications at individual synapses influence the interplay of multiple neurons across a signaling network.

Networks of neurons are studied across many different scales and are generally classified as anatomical, functional, or effective networks depending on the kind of relationship between network nodes that is described by the network edges^17^. Anatomical networks describe the physical connections between brain regions or single cells, as shown for example by diffusion tensor magnetic resonance imaging (MRI)^18–20^, or manual tracings of electron microscopy images^21–24^. Functional networks describe brain regions or cells that are simultaneously active during resting states^25,26^ or a specific task^27,28^ as for example shown by functional MRI, electroencephalography^29,30^, or magnetoencephalography^31,32^. Correlation between time series of the network nodes are used to derive functional networks. Effective networks describe the transmission of information among brain regions or cells as shown for example by multi-electrode array recordings^33–35^, multi-electrode patch clamping^36^, and *in vitro* ^37^ and *in vivo* ^38^ calcium imaging. Causal relations between time series of the network nodes are used to derive effective networks.

On the level of single cells, effective networks represent the actual propagation of action potentials. They are the resulting information processing landscape based on synaptic (e.g. post-synaptic excitability) and cellular (e.g. dendritic spine density) characteristics. While the existence of an effective connection between neurons requires at least one synaptic connection between then, the modification of effective connection strength or the loss of a connection may occur independent of synapse formation and loss: synaptic excitability, size of the active zone, number of synaptic vesicles and post-synaptic receptor density are only some of the many factors that add up to the effective strength of a connection.

How the network interplay of neurons and brain regions is affected in pathological states has become a focus of basic research. For example, altered functional connectivity of brain regions has been reported in the context of Alzheimer’s disease and other types of dementia^39^, and cognitive improvement in Alzheimer’s disease is correlated with a recovery of functional connectivity between the hippocampus and other brain regions^40^. A recent study also shows that amyloid beta peptides, the main components of plaques found in the brains of patients with Alzheimer’s disease, directly modulate neuronal network activity^41^. Furthermore, functional connectivity throughout the brain is impaired in Parkinson’s disease^42^, schizophrenia^43^ and major depressive disorder^44–46^. Effective connectivity has been investigated in the context of epilepsy^35,47^ and Sanfilippo syndrome^48^.

Although previous studies on neuronal silencing have described anatomical changes at the level of single synapses, research on population-wide changes in functional or effective connectivity among neurons has been hindered by the lack of a suitable method of network reconstruction. In the past decade, however, several powerful algorithms, such as mutual information^49^, joint entropy^50^, transfer entropy^51^, and generalized transfer entropy^37^, have been proposed, which can be used to investigate neuronal spike time series and to detect correlations between pairs of them. Here, to assess the effects of neuronal silencing on the connectivity of a neuronal network, we developed a method of reconstructing effective connectivity *in vitro* based on statistical spike time analysis of calcium imaging data using a combination of several powerful algorithms and a machine learning approach for the prediction of interconnected neurons.

## Results

### Neuronal cultures are silenced with tetrodotoxin

**Figure 1:**
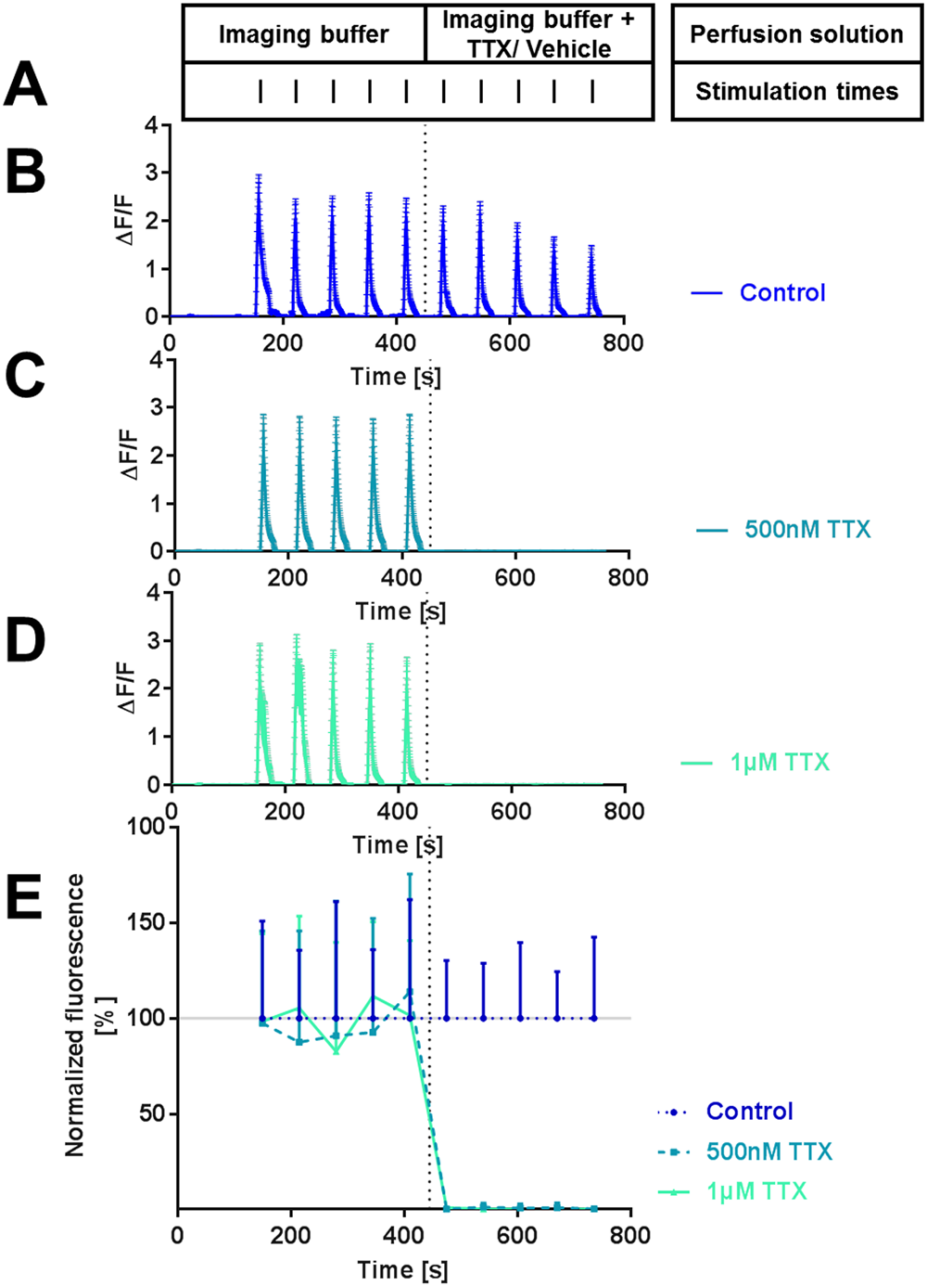
TTX-induced silencing of neuronal responses. TTX blocked neuronal responses as shown by Fluo-4, a calcium-sensitive fluorescent activity marker. Vertical bars indicate time points of electrical stimulation. The dashed line marks the time of perfusion solution switch. (**A**) Scheme of experimental procedure. (**B**–**D**) Representative recording traces (mean with standard deviation). Electrical stimulation with 50 pulses produced robust signals with a high signal-to-noise ratio. (**E**) Time course of Fluo-4 fluorescence signals normalized to the responses in the control condition for each of the 10 pulses (mean and standard deviation). Numbers of analyzed cells with the numbers of independent recordings in parenthesis: control: 399 (6), 500 nM TTX: 571 (8), 1 µM TTX: 644 (11). Similar to those in the connectivity experiments, the cultures were recorded at age DIV 14.

We first tested whether the sodium channel blocker tetrodotoxin (TTX) can silence synaptic activity and thus artificially induce effects of neuronal silencing. We measured the intensity of the fluorescence response upon electrical stimulation of Fluo-4 loaded neurons perfused with imaging buffer supplemented with 500 nM or 1 µM TTX (Fig. 1A,B). The average neuronal response amplitude upon 5 instances of electrical stimulation was reduced to 0.6% of controls ± 0.4% of controls (SD) during perfusion with 500 nM TTX and to 0.1% of controls ± 0.5% of controls (SD) during perfusion with 1 µM TTX (Fig. 1). These results demonstrate that TTX causes synaptic silencing.

**Figure 2:**
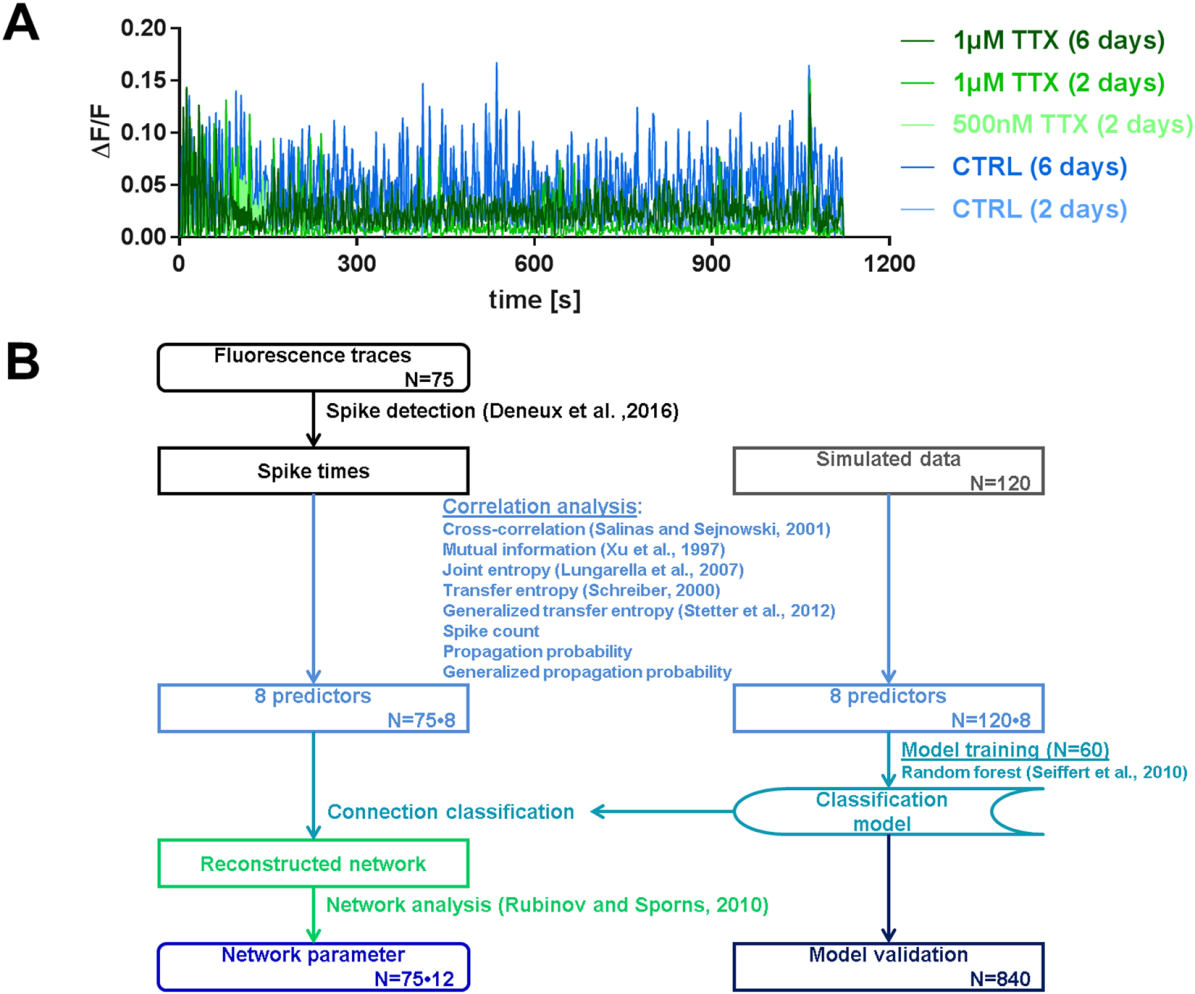
Representative neuronal activity recordings and data processing flow. (**A**) Typical fluorescence time course obtained from Fluo-4 calcium imaging of spontaneous activity of a culture treated with 500 nM TTX, 1 µM TTX or the same volume of the solvent water (control) for two or six days. (**B**) Flow chart of the data processing routine used to reconstruct effective neuronal networks and to obtain network parameters with the number of analyzed recordings and simulations. Annotated names refer to references as cited in the main text.

**Figure 3:**
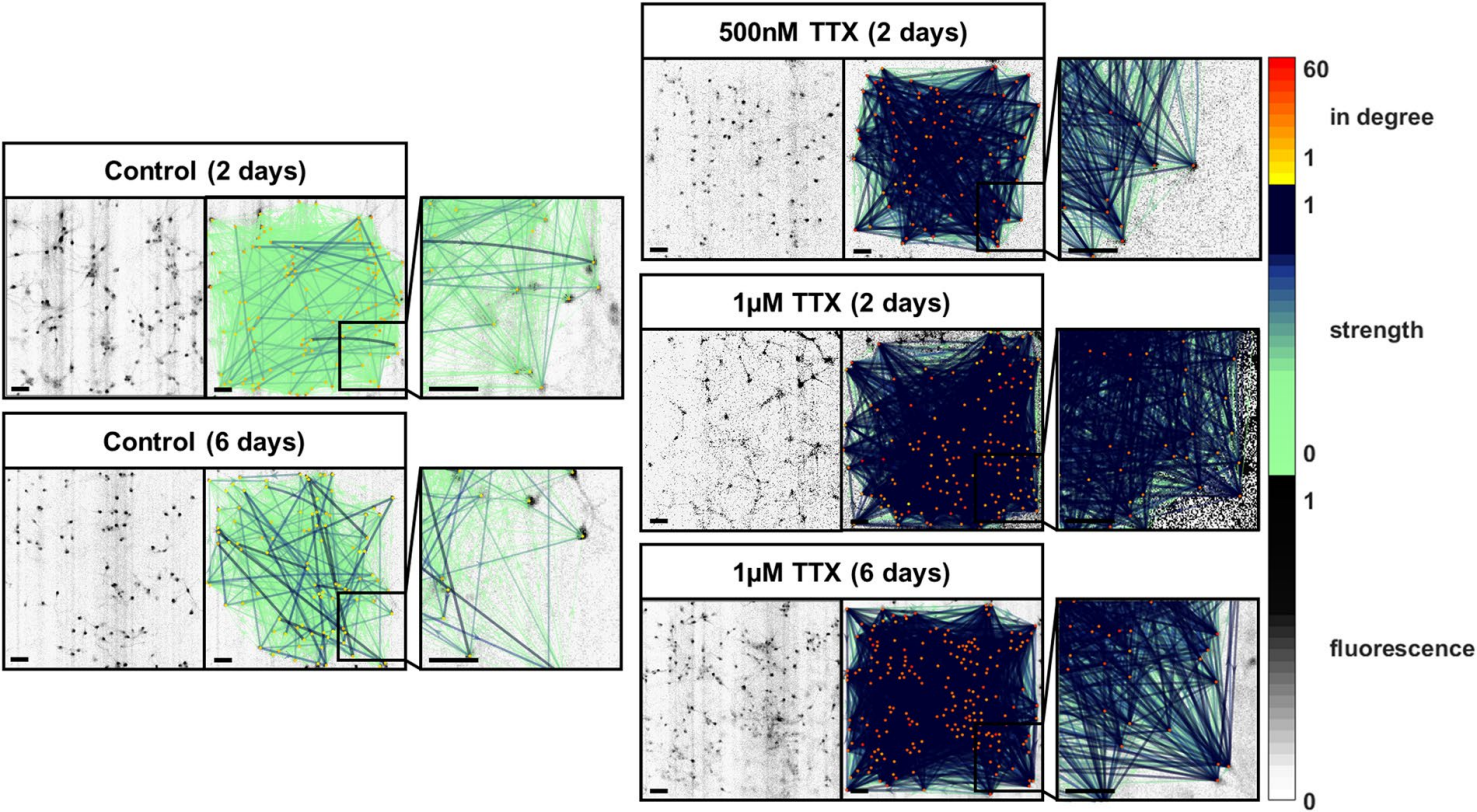
Representative images of effective network reconstruction with connection strengths after TTX-induced silencing for 2 or 6 days. The panels show network plots with color-coded number of incoming connections (in degree) and connection strength (weight). Connection strength is represented by thin, light arrows if low and thick, dark arrows if high. The treatment of neuronal cultures with 500 nM TTX or 1 µM TTX for two or six days yielded effective networks with more connections and higher connection strengths. Scale bars = 100 µm.

To analyze effective connectivity, the culture medium of primary rat hippocampal neurons was supplemented with 500 nM TTX or 1 µM TTX for 2 days or 1 µM TTX for 6 days^7^, and in control cultures the medium was supplemented with respective volumes of the solvent sterile water for the same durations. After incubation, neuronal activity was recorded via calcium imaging (Fig. 2A) and subjected to data processing (Fig. 2B). We performed 60 simulations for training and 840 simulations for testing of the classification model, and we recorded and analyzed a total of 75 experiments across the five treatment groups (Table 1). For details on the simulation model and a validation of the classification model please refer to the supplementary Methods and Supplementary Tables ST1 and ST2. Recording, choice of field of view and data analysis were performed blinded to treatment group. Representative recordings of neuronal cultures and the reconstructed effective networks for all treatment groups are shown in Fig. 3.

**Table 1:** Basic descriptive statistics for all simulations and neuronal recordings.

**Simulations**	
**Classification training data**	
Number of simulations	60
Connectivity degree^3^	20.1% ± 5.0%
Propagation probability^3^	3.9% ± 0.9%
Number of cells per simulation^3^	43 ± 0
**Classification test data**	
Number of simulations	840
Connectivity degree	5–80%
Propagation probability	5–80%
Number of cells per simulation	30–101
**Recordings**	
**Control group, 2 days incubation**	
Treatment	2 days incubation with according volume of water
Number of recordings^1^	11 (12)
Culture age at recording^2^	DIV 12–17
Connectivity degree^3^	21.1% ± 7.0%
Propagation probability^3^	19.6% ± 10.1%
Number of cells per recording^3^	34.9 ± 17.4
**Control group, 6 days incubation**	
Treatment	6 days incubation with according volume of water
Number of recordings^1^	18 (18)
Culture age at recording^2^	DIV 13–15
Connectivity degree^3^	19.6% ± 12.3%
Propagation probability^3^	14.5% ± 5.2%
Number of cells per recording^3^	44.5 ± 13.2
**Silencing group, 2 days incubation**	
Treatment	2 days incubation with 500 nM TTX
Number of recordings^1^	16 (22)
Culture age at recording^2^	DIV 12–17
Connectivity degree^3^	37.2% ± 27.1%
Propagation probability^3^	45.9% ± 21.2%
Number of cells per recording^3^	40.6 ± 21.5
**Silencing group, 2 days incubation**	
Treatment	2 days incubation with 1 µM TTX
Number of recordings^1^	13 (15)
Culture age at recording^2^	DIV (13–15)
Connectivity degree^3^	45.1% ± 20.4%
Propagation probability^3^	37.1% ± 20.5%
Number of cells per recording^3^	43.1 ± 18.6
**Silencing group, 6 days incubation**	
Treatment	6 days incubation with 1 µM TTX
Number of recordings^1^	17 (23)
Culture age at recording^2^	DIV 13–15
Connectivity degree^3^	24.2% ± 15.7%
Propagation probability^3^	34.5% ± 12.1%
Number of cells per recording^3^	49.2 ± 28.5

^1^The numbers of recordings are shown as analyzed recordings (total number of conducted experiments). Recordings with less than 30 active neurons were excluded from the analysis. ^2^DIV = day *in vitro*. ^3^Connectivity degree, propagation probability and number of cells per simulation/ recording are shown as mean ± standard deviation.

### Tetrodotoxin treatment has no effect on the physical distribution of neurons in culture or their viability

Imaged cells were on average 522 µm apart from each another, with no difference in distance between connected and unconnected cell pairs. Also, the strength of formed connections was independent of the connection distance (Supplementary Figure S1). As expected^52^, dissociated hippocampal neurons connected with negative assortativity in all treatment groups (Supplementary Figure S2). The viability of the neuronal cultures did not suffer from the TTX treatment compared to controls (Supplementary Figure S3).

### After neuronal silencing the effective connection strength between neurons is increased

**Figure 4:**
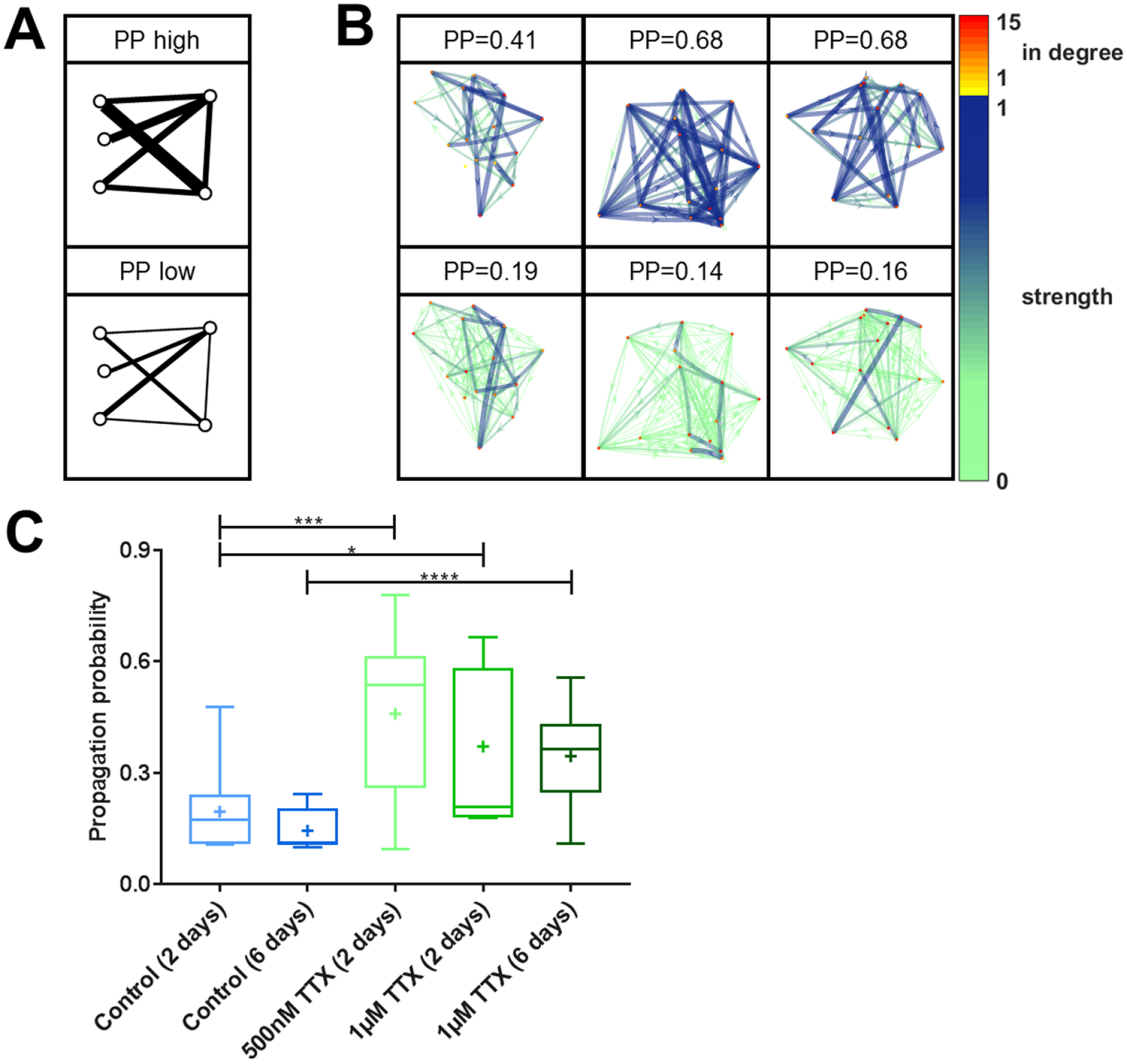
Enhanced propagation probability across a neuronal network after TTX-induced silencing. (**A**) Schematic illustration of high and low propagation probability (PP) in a network. (**B**) Visualization of 15-cell networks with different PP. Weak connections are shown as thin, light arrows, and strong connections are shown as dark, thick arrows. The in-degree – the number of incoming connections - of each node is color-coded. (**C**) After TTX treatment PP is significantly increased (control (2 days) vs. 500 nM TTX (2 days): p = 0.001, control (2 days) vs. 1 µM TTX (2 days): p = 0.019, control (6 days) vs. 1 µM TTX (6 days): p < 0.001, two-sided t-tests). Numbers of experiments: control (2 days): 11, control (6 days): 18, 500 nM TTX (2 days): 16, 1 µM TTX (2 days): 13, 1 µM TTX (6 days): 17. The boxes extend from the 25^th^ to the 75^th^ percentiles. The median and mean are shown as horizontal lines and crosses, respectively. The whiskers show the range of values.

We set out to test, if - additional to the molecular and cellular mechanisms of increased synapse size, vesicular release probability, and neuronal excitability after neuronal inactivity^7^ - there is also an increase in effective connection strength in the neuronal cultures. Using statistical spike time analysis to reconstruct effective connectivity, we examined the probability of propagation of action potentials from one cell to another across the culture (Fig. 4A,B). We found that TTX-induced silencing of neuronal activity for 2 or 6 days increased the propagation probability by approximately 2-fold (Fig. 4C), suggesting that synaptic modifications, such as increased vesicular release probability translate into stronger connections between cells on a population level.

### Neuronal cultures undergo rewiring during activity silencing

**Figure 5:**
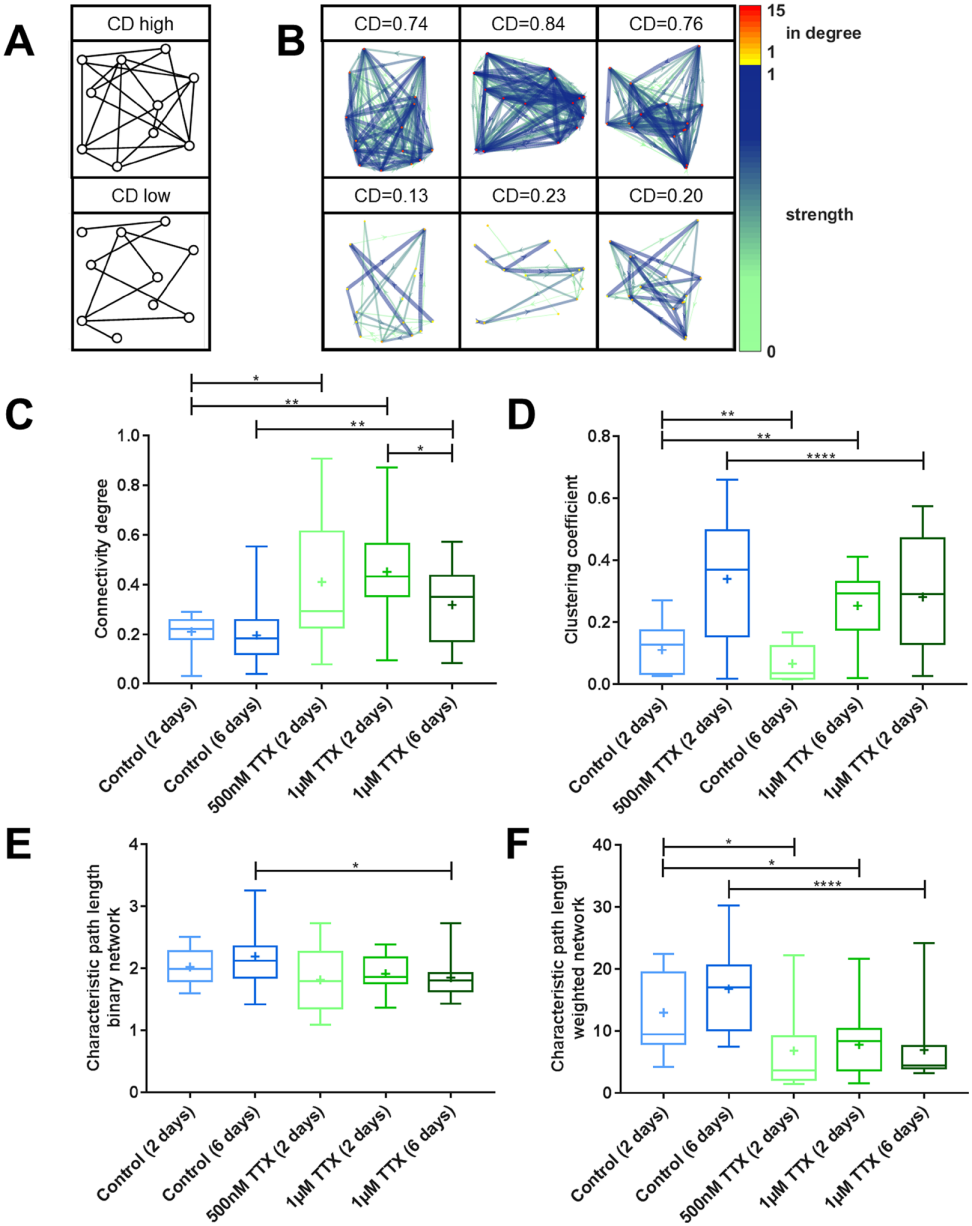
Increased number of connections in neuronal networks after TTX-induced silencing. (**A**) Schematic illustration of networks with high and low connectivity degree (CD), high and low clustering coefficients, and low and high characteristic path length, respectively. (**B**) Visualization of 15-cell networks with different CD. Weak connections are shown as thin, light arrows, and strong connections are shown as dark, thick arrows. The in-degree – the number of incoming connections - of each node is color-coded. (**C**) After TTX treatment the connectivity degree was significantly increased (control (2 days) vs. 500 nM TTX (2 days): p = 0.021, control (2 days) vs. 1 µM TTX (2 days): p < 0.001, control (6 days) vs. 1 µM TTX (6 days): p = 0.010, two-sided t-tests). (**D**) The clustering coefficient was significantly increased after TTX-induced silencing (control (2 days) vs. 500 nM TTX (2 days): p = 0.003, control (2 days) vs. 1 µM TTX (2 days): p = 0.008, control (6 days) vs. 1 µM TTX (6 days): p < 0.001, two-sided t-tests). (**E**) The already low characteristic path length was not significantly lowered after 500 nM or 1 µM TTX treatment for 2 days but after six days of 1 µM TTX treatment (control (2 days) vs. 500 nM TTX (2 days): p = 0.232, control (2 days) vs. 1 µM TTX (2 days): p = 0.364, control (6 days) vs. 1 µM TTX (6 days): p = 0.027, two-sided t-tests). (**F**) After TTX-induced silencing the characteristic path length of the weighted network, which is a combined measure of connection number and connection strength was significantly reduced (control (2 days) vs. 500 nM TTX (2 days): p = 0.032, control (2 days) vs. 1 µM TTX (2 days): p = 0.044, control (6 days) vs. 1 µM TTX (6 days): p < 0.001, two-sided t-tests). Numbers of experiments: control (2 days): 11, control (6 days): 18, 500 nM TTX (2 days): 16, 1 µM TTX (2 days): 13, 1 µM TTX (6 days): 17. The boxes extend from the 25^th^ to the 75^th^ percentiles. The median and mean are shown as horizontal lines and crosses, respectively. The whiskers show the range of values.

When analyzing the structure of the neuronal networks, it appeared not only that the strength of connections between cells was increased but also that the network contained more connections. An additional connection can be established by a synapse formation between two neurons that were not previously synaptically connected. The strengthening of a connection however can be established also by the formation of additional synapses between already connected neurons or by a strengthening of already existing synapses between the two cells. More connections in a network can be quantified in different ways: One parameter is the connectivity degree, which is the number of effective connections normalized to the number of cells in the network. Another parameter, the clustering coefficient, provides information about the distribution of connections. High clustering coefficients appear in networks, in which a cell’s connection partners are also tightly connected with each other (Fig. 5A,B). We found that TTX-induced silencing of neuronal activity for 2 or 6 days approximately doubled both the connectivity degree and the clustering coefficient (Fig. 5C,D).

A third way to characterize the number of connections of a network is the characteristic path length, which describes the average distance between two cells in a network (with “distance” being defined as the sum of intermediate steps in the shortest path between cells). In a network with many connections, paths between two cells tend to be relatively direct, resulting in a short characteristic path length. In a more sparsely connected network, however, the most direct path between two cells requires a number of intermediate steps, resulting in a longer characteristic path length. Details and formulas to calculate the topology measures can be found in the Supplementary Methods. This characteristic path length of the control networks were 2.02 (mean of 2 day controls ± 0.28 standard deviation) and 2.19 (mean of 6 day controls ± 0.50 standard deviation), which indicates a network in which almost every cell is directly connected to every other cell (characteristic path length of 1). The additional connections in TTX treated cultures as seen in the connectivity degree did form at sites in the network that only slightly decreased the characteristic path length (Fig. 5E). When investigating effective (or functional) networks one can also calculate the weighted characteristic path length^53^. This measure represents also the sum of intermediate steps of the shortest paths in the network but each intermediate connection is weighted by the respective connections strength. The weighted characteristic path length represents a combined measure of connection number and connection strengths. We found that TTX-induced silencing of neuronal activity for 2 or 6 days reduced the weighted characteristic path length by approximately half (Fig. 5F), demonstrating the extent of rewiring and connection strengthening during neuronal silencing.

### Transmission efficiency is enhanced in tetrodotoxin treated neuronal cultures

**Figure 6:**
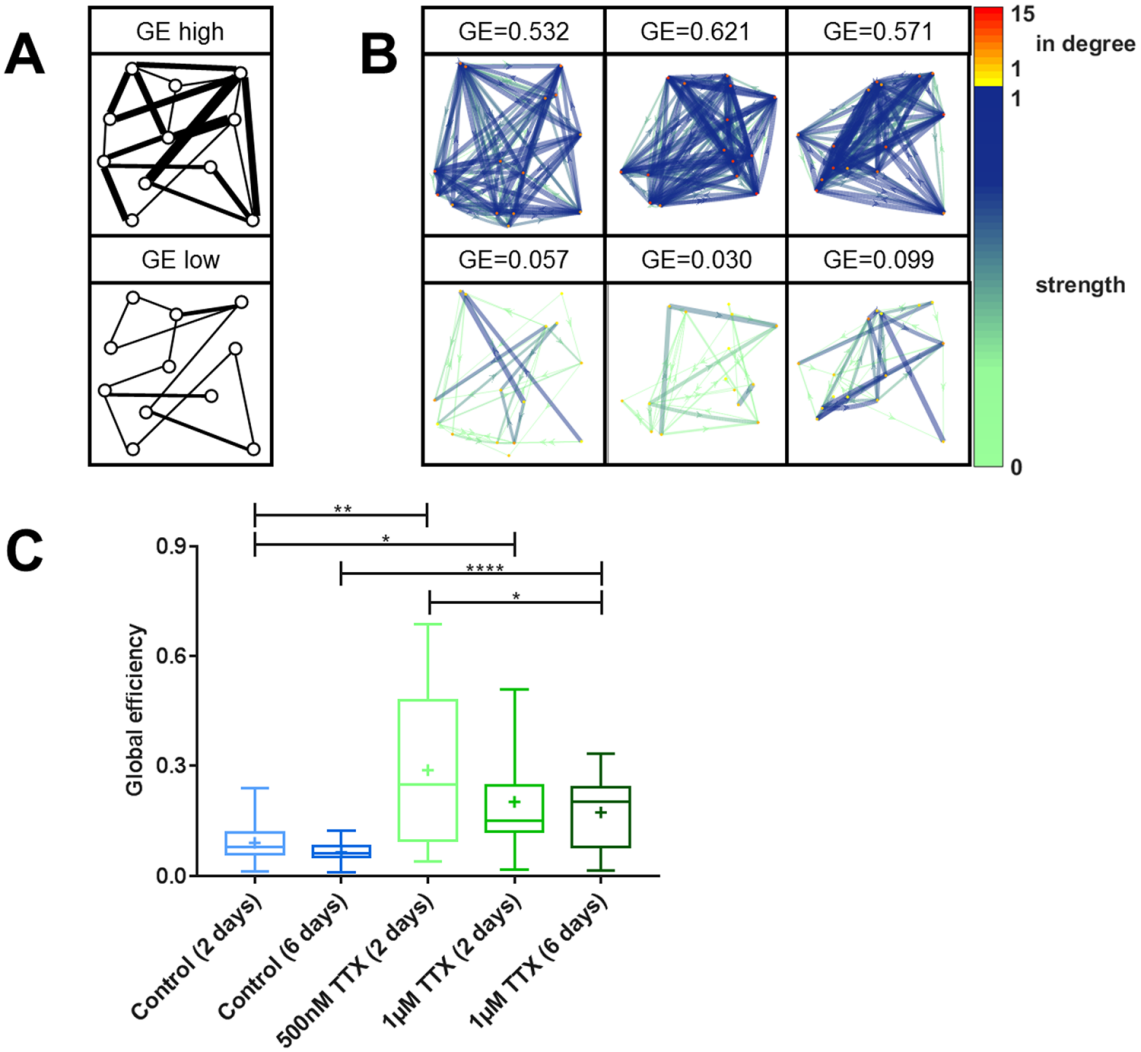
Potentiated signal transmission efficiency after TTX-induced silencing. (**A**) Schematic illustration of networks with high and low global efficiency (GE). (**B**) Visualization of 15-cell networks with different GE. Weak connections are shown as thin, light arrows, and strong connections are shown as dark, thick arrows. The in-degree – the number of incoming connections - of each node is color-coded. (**C**) After TTX treatment GE was significantly enhanced (control (2 days) vs. 500 nM TTX (2 days): p = 0.007, control (2 days) vs. 1 µM TTX (2 days): p = 0.027, control (6 days) vs. 1 µM TTX (6 days): p < 0.001, two-sided t-tests). Numbers of experiments: control (2 days): 11, control (6 days): 18, 500 nM TTX (2 days): 16, 1 µM TTX (2 days): 13, 1 µM TTX (6 days): 17. The boxes extend from the 25^th^ to the 75^th^ percentiles. The median and mean are shown as horizontal lines and crosses, respectively. The whiskers show the range of values.

Using the method of reconstructing the effective network of an entire population of neurons, we observed stronger connections and an increased number of connections after the silencing, resulting in a network twice as dense with effective connections as its original state. The outcome of these two processes can be quantified as the signal transmission efficiency across the network^53^ (Fig. 6A,B). This measure describes how the combination of number of connections and their individual strengths combine to form the actual information processing landscape of the network. We found that the combination of both silencing effects boosted the global transmission efficiency by nearly 4-fold (Fig. 6C). Such a potentiation of synaptic communication in previously silenced neuronal cultures may most likely be an effect of increased action potential propagation probability and possibly the growth of new connections.

Taken together, these results show that the silencing of neuronal activity not only increases action potential propagation across connections but also leads to the formation of many additional effective connections.

## Discussion

Our observations confirm previous reports that the silencing of neuronal activity over a short time span potentiates the strength of synaptic connections. However, whereas earlier studies only show that neuronal inactivity induces changes at the level of individual synapses, including increased synapse size and postsynaptic receptor concentration^5–7^, our study demonstrates that neuronal silencing increases action potential propagation probability across an entire neuronal network. In addition, we discovered reduced neuronal activity not only increased synaptic sensitivity^7^ but also a largely increased number of connections throughout the network.

The ability to detect differences in connectivity patterns is an important first step towards understanding how unused neurons become reconnected, such as during the recovery of stroke patients. Research in this area has generated an extensive body of evidence supporting the hypothesis that recovery from stroke reflects the recovery of connectivity of “silenced” brain regions^54–59^. A deeper understanding of this process could help to accelerate the recovery of functional connections not only in stroke patients but also in those with spatial neglect^60,61^, traumatic brain injury^62,63^, or glioma^64^. A deeper knowledge of this rewiring process also provides new insight into the sprouting of new dendritic spines during neuronal silencing^65^, which has long been observed but not explained.

Neuronal cultures, silenced for 6 days, exhibited reduced strengthening and formation of synaptic connections and less potentiation of synaptic transmission compared with those silenced for 2 days with the same TTX concentration of 1 µM. This suggests that the potentiating effects of neuronal silencing compete against the opposing loss of connections, leading to the weakening and ultimately to the loss of effective connections with more prolonged inactivity.

These effects occurred without an impairment of cell viability after the pharmacological treatment (Supplementary Figure S3). Also the overall synchrony of the neuronal activity in the culture was unaffected by the treatment (Supplementary Figure S4). The causal relation between different cells’ time series that we measured as effective connection strengths, however, showed the reported increase after TTX treatment . Additional evidence supporting the hypothesis of competing connection strengthening and connection loss comes from a more detailed analysis of neuronal network structure. With the disappearance of alternative routes of communication among cells after 6 days of TTX-induced silencing, the betweenness centrality of cells with remaining connections increased (Supplementary Figure S5). The number of incoming and outgoing connections, which is increased after two days of silencing normalized back to the original values after six days of treatment with the same TTX concentration (Supplementary Figure S6). This is in line with the previously described reduction of dendritic arborization and length after longer-term synaptic inactivity^66^. Understanding how to shift this counterbalance toward the densification of neuronal networks and the strengthening of connections might facilitate the development of treatment options for a wide range of diseases such as multiple sclerosis^67,68^, Alzheimer’s disease^39,41,69^, mood disorders^70–72^, and addiction^73^.

Although neurons may have appeared to make indiscriminant connections to retain synaptic transmission during network silencing, the connections were not formed at random. Rather, the maintenance of a constant out-per-in-degree and modularity during neuronal inactivity (Supplementary Figure S7) point toward an action potential-independent regulatory mechanism. A previous study shows that neurons maintain their distribution of different synapse types under TTX treatment^66^. Such basic network properties could be maintained in the absence of neuronal firing through signaling between axons and synaptic terminals or communication via spontaneous synaptic vesicle release^74,75^, although further research on this topic is needed.

In summary, using a novel methodological approach, we uncovered the formation of new effective connections during short-term (2 days) but not long-term (6 days) silencing of neuronal activity. This approach opens the field for further research on population-wide effects of different pharmacological substances and implicates neuronal connectivity as a possible drug target in neurological and psychiatric diseases.

## Materials and Methods

### General methods

We followed the Standards for Reporting Diagnostic Accuracy Studies (STARD)^76^ adapted to our study design in accordance with recommendations for good scientific practice by the German Research Foundation^77^. All chemicals were obtained from Invitrogen (Carlsbad, CA) unless stated otherwise. The incubation wells for the different experimental groups were randomly assigned and imaging and image processing were conducted blinded to the experimental condition to avoid expectation bias when choosing a field of view for imaging or parameters for data analysis.

### Code availability

Image processing and analysis, as well as network reconstruction and analysis, were carried out using original scripts in MATLAB (RRID: SCR_001622, Mathworks Inc., Natick, MA). Graph theoretical network analysis was performed using the subfunctions provided in the Brain Connectivity Toolbox by Rubinov *et al*. (RRID: SCR_004841)^53^. All scripts used to generate data are available in the GitHub repository (www.github.com/janawrosch/effective_connectivity). The box-and-whishkers plots were generated with the Graph Pad Prism software (La Jolla, CA).

### Data availability

Detailed tables of the data and interferential statistics can be found in the Supplementary Excel-Item. Other data are available from the corresponding author per request at any time.

### Cell culture

Primary hippocampal neurons were obtained as previously described^78,79^. Briefly, hippocampi were removed from 1- to 3-day-old Wistar rats of any sex (RRID: RGD_68115, Charles River, Wilmington, MA) after sacrifice in accordance with guidelines of the State of Bavaria and with approval by the ethics committee of the Friedrich-Alexander University of Erlangen-Nürnberg. Hippocampal cells were washed, digested, dissociated, and centrifuged. The cell pellet was resuspended in medium, and cells were plated on glass coverslips in a 12-well plate coated with Matrigel^TM^ (BD Biosciences, San Jose, CA). After a medium change on day 2 *in vitro* (DIV 2), cells were incubated until use in experiments. We conducted 75 experiments with 75 different coverslips that were seeded in 43 independent preparations. The preparations were conducted on different days from groups of 6 animals, each.

### Pharmacological treatments

Experimental cell cultures were treated with 500 nM or 1 µM TTX (Sigma-Aldrich, CAS 4368-28-9, St. Louis, MO) for 2 days or with 1 µM TTX for 6 days. TTX was diluted in sterile water. Control cell cultures were treated with the respective volumes of sterile water for the same durations. These TTX concentrations and incubation times were used in a previous major study of synaptic disuse^7^. The culture medium was supplemented with TTX or sterile water on the according number of days before the imaging experiments, which were conducted on DIV 12–17.

### Cell viability: LDH-assay

Cell viability after the pharmacological treatments was assessed with a lactate-dehydrogenase-assay (Promega Cyto Tox 96 Non-Radioactive Cytotoxicity Assay (Madison, WI)) according to manufacturer instructions: The supernatant of 5 different cultures was collected for each condition. Additionally, the supernatant of two different lysed cultures and a sample of pure medium were collected. The samples were analyzed in triplicates. Each sample was incubated with the same volume of substrate-buffer-mix (containing lactate and tetrazolium) for 30 minutes at room temperature and the reaction was stopped with the same volume of stop-solution. The color change yielded by the conversion of tetrazolium to red formazan with NADH available from the enzymatic activity of lactate-dehydrogenase was quantified with a BioRad Benchmark Photometer (Hercules, CA) at 490 nm in a flat-bottom 96-well plate three times and averaged. The ratio of cytotoxicity was calculated according to manufacturer instructions:

$$[ \% \,cytotoxicity]=\frac{[measured\,LDH\,release]}{[maximum\,LDH\,release\,(lysis)]}\cdot 100$$

### Live cell recording of neuronal activity for network reconstruction

To ensure adequate cell health, samples were chosen after bright field microscopy inspection, excluding coverslips that showed signs of weak vitality (e.g., no dense soma or detached, loose axons). Detailed descriptions of the staining process and the experimental setup are provided in the Supplementary Methods. Briefly, cultures were stained with the calcium-sensitive fluorescent dye Fluo-4-AM, washed with phosphate-buffered saline, and placed into an imaging chamber filled with imaging buffer. Recordings were made at room temperature on a Nikon TI-Eclipse inverted fluorescence microscope equipped with a 10×, 0.45 NA objective (Nikon Instruments Europe, Düsseldorf, Germany) and a water-cooled EM-CCD camera (iXon Ultra 897, Andor, Belfast, Northern Ireland). For each recording, we captured 18.5 min of spontaneous activity, which is sufficient to allow network reconstruction with maximal accuracy (Supplementary Figure S8A). After recording spontaneous activity, we delivered a 1200-pulse electrical field stimulation train of 40 Hz for the automatic detection of responsive regions of interest, delivered at alternating polarity through two parallel platinum electrodes spaced 10mm apart. Images were recorded using Andor Solis software with an exposure time of 1 ms and a recording frame rate of 27.33 Hz, resulting in 31156 frames per recording. Recordings were exported into tagged image file format containing 512 × 512 pixels of 16-bit monochromatic intensity values.

### TTX-induced silencing of neuronal activity

To achieve neuronal silencing through TTX application, cells were prepared as described above and imaged using the same experimental setup with the addition of a fast-step perfusion system (Warner Instruments, Hamden, CT). After cells acclimatized to perfusion for 2:30 min, they were repeatedly stimulated with 10 trains of 50 1-ms pulses of alternating polarity at 10 Hz with a 60-s inter-stimulation interval. During the first phase of the experiment (i.e., the first five stimulations, 445 s), cells were perfused with plain imaging buffer. During the second phase of the experiment (i.e., the second five stimulations, 325 s), cells were perfused with imaging buffer supplemented with 500 nM TTX, 1 μM TTX, or equivalent volumes of sterile water. We recorded the fluorescence increase of the cells in response to the electrical stimulations. Each cell’s ten response amplitudes were normalized to the average response amplitudes in the control condition.

### Image processing and network reconstruction

A detailed description of the image processing and network reconstruction steps is provided in the Supplementary Methods. Briefly, images were filtered according to the fluorescence increase upon electrical stimulation, ensuring the detection of only viable neuronal cells, and regions of interest (i.e., neuronal cell bodies) were detected using a feature point detection algorithm^80^. The fluorescence signal was extracted from the image stacks and the relative fluorescence ΔF/F was derived as previously described^81^. Underlying action potentials were inferred from the relative fluorescence traces using an extended template fitting spike estimation algorithm^82^. The gained spike trains for each cell were analyzed to determine similarity and causal relations between all possible pairs of cells with eight different methods: simple cross-correlation^83^, mutual information^49^, joint entropy^50^, transfer entropy^51^, generalized transfer entropy^37^, spike count, propagation probability and generalized propagation probability. A detailed description of the used algorithms can be found in the Supplementary Methods. The resulting eight values for each pair of cells were then used as predictors in a random undersampled adaptive boosted random forest classification model^84^ to determine which cell pair’s causal links were strong enough to be classified as connected. This classification may err so the sensitivity, specificity and accuracy of the classification were validated on simulations with a wide range of initial parameters and the same time resolution as the *in vitro* imaging data (Supplementary Table ST1). On simulated test data with parameters similar to those of the classification training data the model classified with an accuracy of 91.1%, sensitivity of 77.8% and specificity of 93.3% (Supplementary Figure 8C).

### Network analysis

Reconstructed effective networks were analyzed using the predicted connection strength as the connection weight. From a multitude of possible network topology properties^53^, we chose to analyze 12 different features that can be transferred to an underlying biological rationale. Detailed mathematical descriptions of the network topology parameters are found in the Brain Connectivity Toolbox documentation by Rubinov *et al*. (RRID: SCR_004841)^53^ and in the Supplementary Methods. Detailed descriptive and inferential statistics for each network parameter are provided as the Tables in the Supplementary Excel-Item.

## Electronic supplementary material

Supplementary Material

Supplementary Data File
