# Supplementary material for: Rewiring of neuronal networks during synaptic silencing
Source: Sci Rep. 2017 Sep 15;7:11724. doi: 10.1038/s41598-017-11729-5 (PMC5601899; doi:10.1038/s41598-017-11729-5)
Supplement: Supplementary file 1 — Supplementary Material [file 41598_2017_11729_MOESM1_ESM.pdf]

Rewiring of neuronal networks during synaptic silencing

Jana Katharina Wrosch, Vicky von Einem, Katharina Breininger, Marc Dahlmanns, Andreas Maier, Johannes Kornhuber, Teja Wolfgang Groemer

Supplementary Figures and Tables

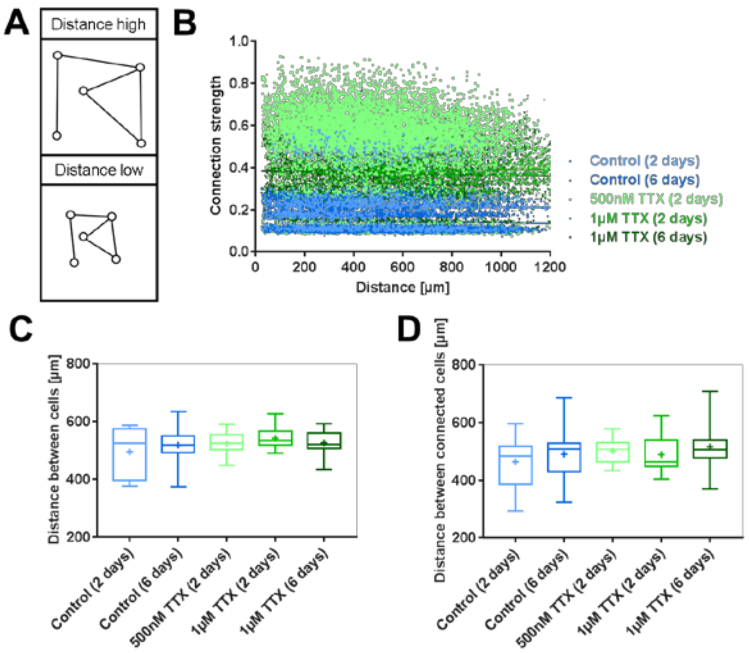

Supplementary Figure S1. Connection distance between cells in TTX-treated cultures. (A) Illustrations of networks with low and high physical distance between cells. (B) The connection strength was independent of the distance between connecting cells. Linear regression slope did not significantly differ from zero for any treatment group (control (2 days): p = 0.513, control (6 days): p = 0.625, 500nM TTX (2 days): p = 0.166, 1µM TTX (2 days): p= 0.394, 1µM TTX (6 days): p = 0.222). Number of connections: control (2 days): 2161, control (6 days): 6608, 500nM TTX (2 days): 11820, 1µM TTX (2 days): 7611, 1µM TTX (6 days): 7613. (C) In our experiment, the distance between all recorded cells and (D) connected pairs of cells was comparable across all treatment groups (all not significant in two-sided t-tests). Numbers of experiments: control (2 days): 11, control (6 days): 18, 500nM TTX (2 days): 16, 1µM TTX (2 days): 13, 1µM TTX (6 days): 17. The boxes extend from the 25th to the 75th percentiles. The median and mean are shown as horizontal lines and crosses, respectively. The whiskers show the range of values.

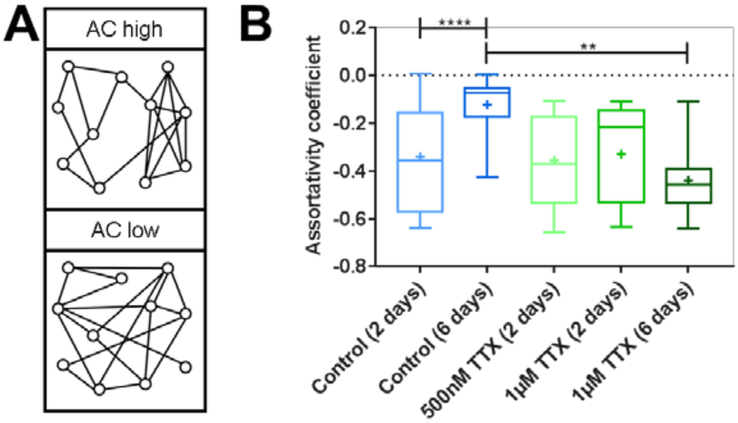

Supplementary Figure S2. Negative assortativity in effective networks of dissociated rat hippocampal neurons. Assortativity coefficients (AC) were calculated based on the in-degree/in-degree correlation of connected cells and are a measure of preferential attachment. (A) Positive values indicate an attachment of cells to other cells with a similar number of connections, whereas negative values indicate an attachment of cells to other cells with a different number of connections. As random connections are equally frequent between similar and dissimilar cells, the average assortativity coefficient is near zero. (B) There were significant differences between the two control groups ($p = 0.002$) and between the control (6 days) and 1µM TTX (6 days) groups ($p < 0.001$, two-sided t-tests). Numbers of experiments: control (2 days): 11, control (6 days): 18, 500nM TTX (2 days): 16, 1µM TTX (2 days): 13, 1µM TTX (6 days): 17. The boxes extend from the 25th to the 75th percentiles. The median and mean are shown as horizontal lines and crosses, respectively. The whiskers show the range of values.

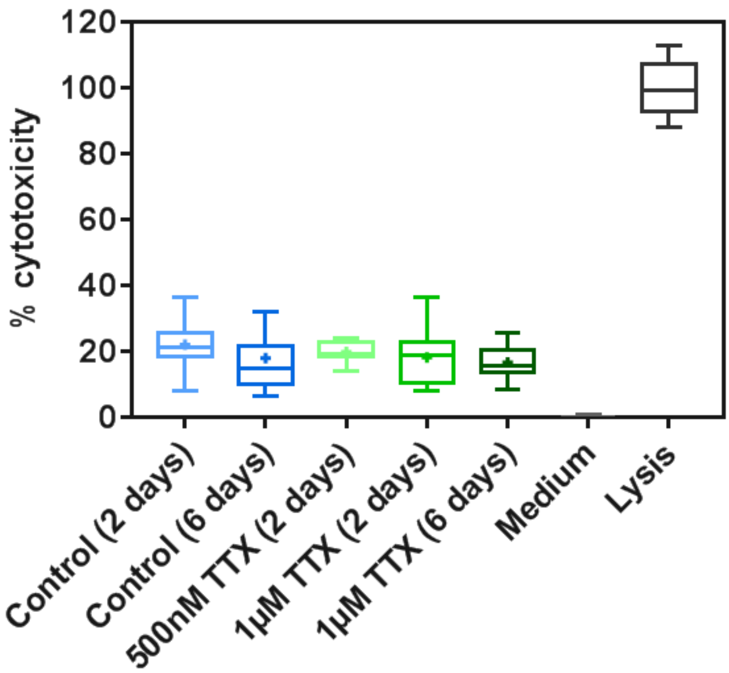

Supplementary Figure S3. Cytotoxicity of TTX treated cultures compared to controls. Cytotoxicity was assessed with an LDH-assay. The average background values of medium without cells was set to zero, the average maximum LDH-release from lysed cells was set to 100% cytotoxicity. The cultures were treated prior to the experiment with 500nM TTX for 2 days, 1µM TTX for 2 days, 1µM TTX for 6 days or with the respective volume of the vehicle water from 2 days or 6 days. The data was recorded from 27 independent cultures (5 per condition + 2 for lysis) that were each measured in triplicates. Medium background was also measured as a triplicate. LDH-substrate absorbance was measured at 490nm three times and the results were averaged. The age of the used cultures was DIV 16 – similar to that of the cultures used for the network analysis recordings. The boxes extend from the 25th to the 75th percentiles. The median and mean are shown as horizontal lines and crosses, respectively. The whiskers show the range of values.

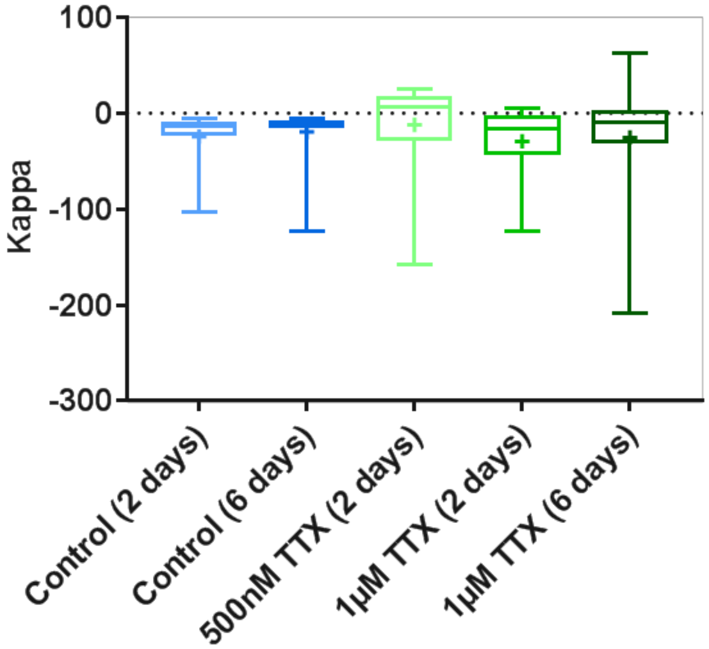

Supplementary Figure S4. Synchrony of neuronal activity in cultures treated with TTX for two and six days. Kappa, a measure of activity synchrony between the recorded cells was not significantly affected by the treatment with 500nM TTX for two days (p=0.425), with 1µM TTX for two days (p=0.724) or six days (p=0.744). The boxes extend from the 25th to the 75th percentiles. The median and mean are shown as horizontal lines and crosses, respectively. The whiskers show the range of values.

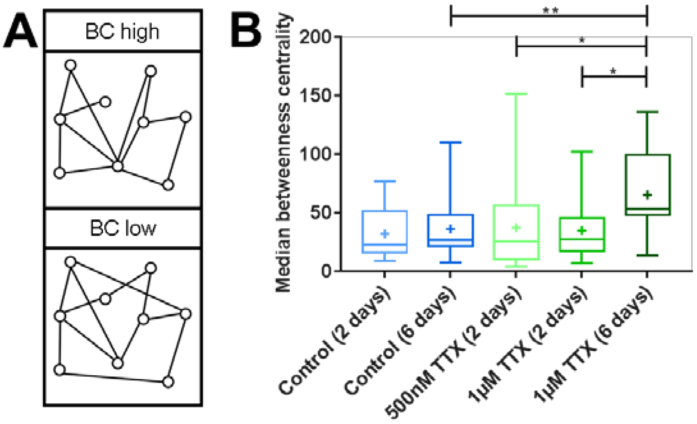

Supplementary Figure S5. Betweenness centrality during 2 days and 6 days of TTX-induced silencing. Betweenness centrality (BC) describes the number of shortest and most efficient paths through the network that include a given node. (A) Networks with high betweenness centrality route their signals through central bottleneck cells, whereas networks with low betweenness centrality use more distributed signaling routes. (B) During 2 days of TTX-induced silencing, the naturally low betweenness centrality in the neuronal cultures was maintained (control (2 days) vs. 500nM TTX (2 days): p = 0.694, control (2 days) vs. 1µM TTX (2 days): p=0.781, two-sided t-tests). During 6 days of TTX-induced silencing, alternative routes dissipated, and betweenness centrality increased (control (6 days) vs. 1µM TTX (6 days): p=0.005, 1µM TTX (2 days) vs. 1µM TTX (6 days) p=0.011, two-sided t-tests). Numbers of experiments: control (2 days): 11, control (6 days): 18, 500nM TTX (2 days): 16, 1µM TTX (2 days): 13, 1µM TTX (6 days): 17. The boxes extend from the 25th to the 75th percentiles. The median and mean are shown as horizontal lines and crosses, respectively. The whiskers show the range of values.

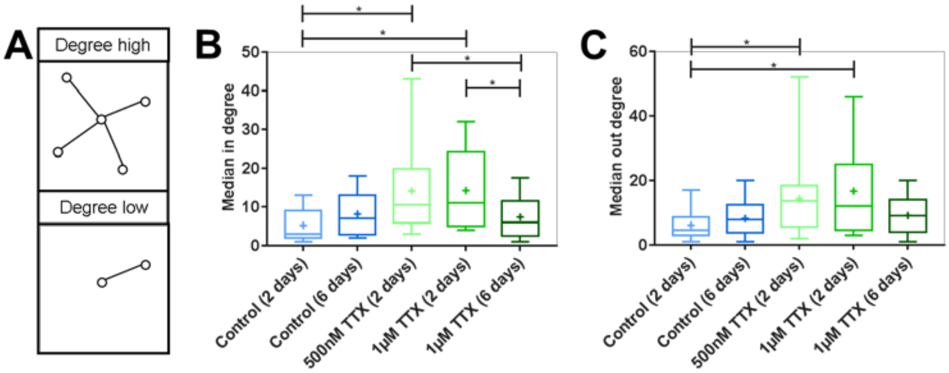

Supplementary Figure S6. Neuronal cultures' in- and out-degree after TTX-induced silencing. The in- and out-degree describe the number of incoming and outgoing connections of a given node. (A) Illustrations of networks with low and high degree. The average number of (B) incoming and (C) outgoing connections per cell increased after 2 days but not after 6 days of TTX-induced silencing (in degree: control (2 days) vs. 500nM TTX (2 days): p = 0.019, control (2 days) vs. 1µM TTX (2 days): p=0.012, control (6 days) vs. 1µM TTX (6 days): p = 0.660, 1µM TTX (2 days) vs. 1µM TTX (6 days): p = 0.022; out degree: control (2 days) vs. 500nM TTX (2 days): p = 0.042, control (2 days) vs. 1µM TTX (2 days): p=0.035, control (6 days) vs. 1µM TTX (6 days): p = 0.652, 1µM TTX (2 days) vs. 1µM TTX (6 days): p = 0.062; two-sided t-tests). Numbers of experiments: control (2 days): 11, control (6 days): 18, 500nM TTX (2 days): 16, 1µM TTX (2 days): 13, 1µM TTX (6 days): 17. The boxes extend from the 25th to the 75th percentiles. The median and mean are shown as horizontal lines and crosses, respectively. The whiskers show the range of values.

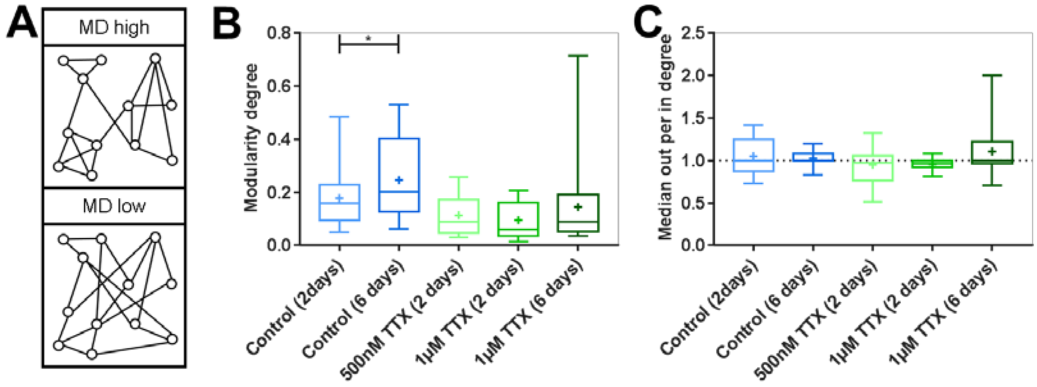

Supplementary Figure S7. Basic network topology in effective networks of neurons after inactivity-induced rewiring. Modularity degree (MD) is the degree to which a network can be subdivided into compact modules of cells that are highly inter-connected and sparsely intra-connected or consists of connections that are evenly distributed. The out per in degree describes whether the nodes of a network are rather converging (many incoming, little outgoing connection – low in per out degree) or diverging (little incoming, many outgoing connections – high in per out degree). (A) Illustrations of networks with low and high modularity degree. The (B) modularity degree and (C) ratio of outgoing to incoming connections per cell were mostly maintained during TTX-induced silencing (modularity degree: control (2 days) vs. 1µM TTX (2 days): p=0.044, all others: not significant, two-sided t-tests). Numbers of experiments: control (2 days): 11, control (6 days): 18, 500nM TTX (2 days): 16, 1µM TTX (2 days): 13, 1µM TTX (6 days): 17. The boxes extend from the 25th to the 75th percentiles. The median and mean are shown as horizontal lines and crosses, respectively. The whiskers show the range of values.

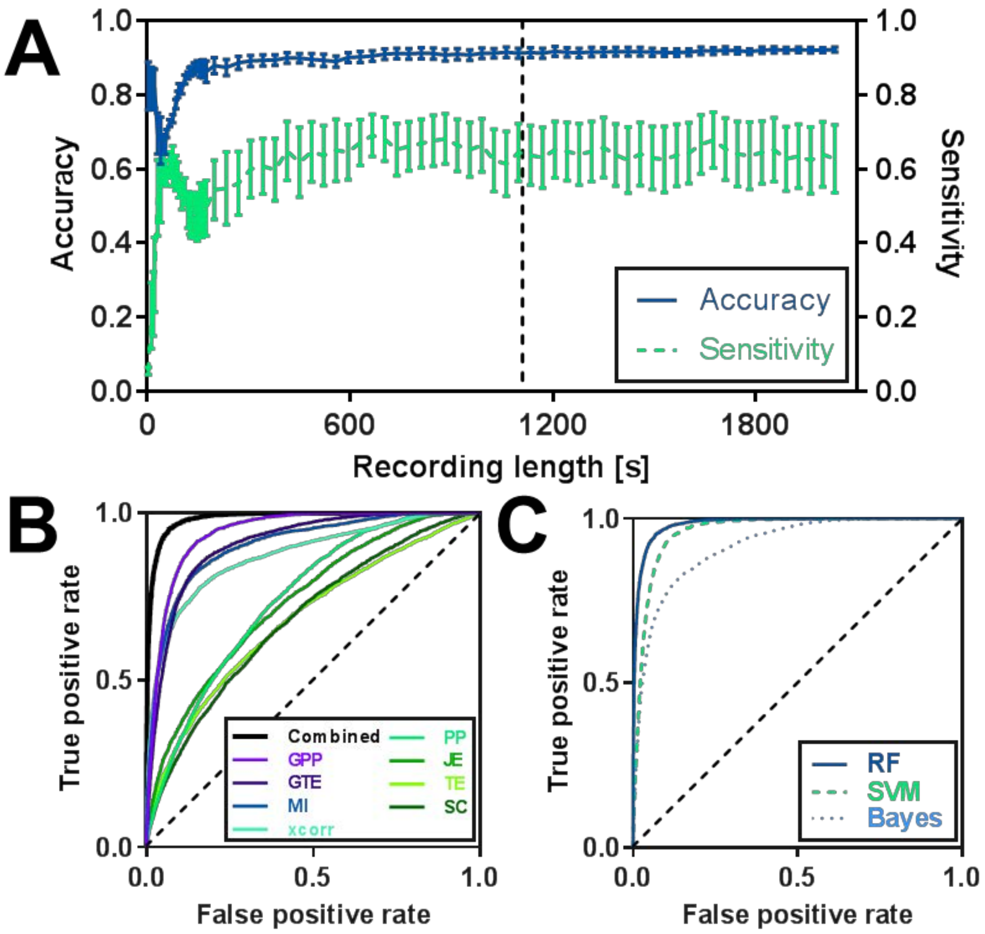

Supplementary Figure S8. Estimation of recording length, predictors and classification model for the inference of effective networks from calcium imaging data. (A) With more data, the network reconstruction (i.e., classification of connected and unconnected cell pairs) becomes more accurate and sensitive. The dashed line indicates the used recording length of 18.5 min, which corresponds to approximately 600 spikes per cell in untreated cultures. The plot shows the mean with S.E.M. of classification accuracy (blue solid line) and sensitivity (green dashed line) of the used classifier in five simulations. (B) The combination of eight correlation measures outperformed the predictive performance of only one measure at a time. The plot shows a receiver operating characteristics curve of the classification of connected cells in the network based on each correlation measure separately versus all eight correlation measures combined. Youden indices for the classification based on different predictors: combined: 0.828, generalized propagation probability (GPP): 0.706, generalized transfer entropy (GTE): 0.663, mutual information (MI): 0.632, cross correlation (xcorr): 0.602, propagation probability (PP): 0.344, joint entropy (JE): 0.325, spike count (SC): 0.262, transfer entropy (TE): 0.261, all eight predictors combined: 0.828. The analysis was performed using training and testing data sets of 60 simulations each with equal prior class probabilities, each. (C) The random forest classification model outperformed other common classifiers in predicting effective connections in simulations of calcium imaging recordings. Receiver operating characteristic curves show the results of 10-fold inner cross-validation on a test data set of 60 simulations. Classifiers were trained on another 60 simulations with equal prior class probabilities as the test data after random undersampling of the data set. Parameter of Random Forest (RF): 600 weak learners, adaptive boosted, 0.1 learning rate, minimum leaf size of 5; Youden index: 0.828. Parameter of Support Vector Machine (SVM): box-constrained linear SVM with constant misclassification cost; Youden index: 0.817. Parameter of naïve Bayes (Bayes): Gaussian-distributed kernel smoothing with unbounded density support; Youden index: 0.686.

Supplementary Table ST1. Validation of classification model for predicting effective connections.

	CD=5%	CD=10%	CD=20%	CD=40%	CD=60%	CD=80%
PP=5%	Sensitivity=0.0% *	Sensitivity=0.0% *	Sensitivity=77.8%	Sensitivity=99.9%	Sensitivity=99.9%	Sensitivity=99.9%
	Specificity=100.0% *	Specificity=100.0% *	Specificity=93.3%	Specificity=100.0%	Specificity=100.0%	Specificity=100.0%
	Accuracy=95.2% *	Accuracy=90.7% *	Accuracy=91.1%	Accuracy=99.9%	Accuracy=99.9%	Accuracy=99.9%
PP=10%	Sensitivity=99.1%	Sensitivity=99.6%	Sensitivity=99.5%	Sensitivity=98.3%	Sensitivity=98.7%	Sensitivity=99.1%
	Specificity=100.0%	Specificity=100.0%	Specificity=100.0%	Specificity=99.6%	Specificity=99.5%	Specificity=99.6%
	Accuracy=100.0%	Accuracy=100.0%	Accuracy=99.9%	Accuracy=99.2%	Accuracy=99.2%	Accuracy=99.3%
PP=20%	Sensitivity=100.0%	Sensitivity=99.6%	Sensitivity=98.9%	Sensitivity=99.9%	Sensitivity=99.7%	Sensitivity=99.6%
	Specificity=100.0%	Specificity=100.0%	Specificity=99.8%	Specificity=100.0%	Specificity=99.9%	Specificity=99.7%
	Accuracy=100.0%	Accuracy=100.0%	Accuracy=99.6%	Accuracy=99.9%	Accuracy=99.8%	Accuracy=99.7%
PP=40%	Sensitivity=99.1%	Sensitivity=98.4%	Sensitivity=98.9%	Sensitivity=97.6%	Sensitivity=93.9%	Sensitivity=91.0%
	Specificity=100.0%	Specificity=99.9%	Specificity=99.7%	Specificity=98.9%	Specificity=95.6%	Specificity=89.7%
	Accuracy=99.9%	Accuracy=99.7%	Accuracy=99.6%	Accuracy=98.4%	Accuracy=94.9%	Accuracy=90.4%
PP=60%	Sensitivity=95.3%	Sensitivity=97.0%	Sensitivity=91.7%	Sensitivity=80.3%	Sensitivity=70.3%	Sensitivity=70.7%
	Specificity=99.6%	Specificity=99.4%	Specificity=98.4%	Specificity=90.9%	Specificity=80.3%	Specificity=70.1%
	Accuracy=99.4%	Accuracy=99.2%	Accuracy=97.2%	Accuracy=87.5%	Accuracy=75.9%	Accuracy=70.4%
PP=80%	Sensitivity=85.0%	Sensitivity=81.3%	Sensitivity=72.2%	Sensitivity=60.8%	Sensitivity=49.7%	Sensitivity=47.0%
	Specificity=99.1%	Specificity=97.5%	Specificity=91.1%	Specificity=77.8%	Specificity=66.4%	Specificity=60.1%
	Accuracy=98.4%	Accuracy=95.9%	Accuracy=87.7%	Accuracy=72.3%	Accuracy=58.9%	Accuracy=53.0%

The classification model prediction sensitivity, specificity and accuracy for each parameter combination of propagation probability (PP) and connectivity degree (CD) were assessed on the basis of 20 simulations, each. All simulations were processed with the same algorithms as the in vitro recordings and classification performance was calculated by comparison to the ground truth given by the simulated connections and averaged. The number of cells was 43 – the average of all performed in vitro recordings – in all simulations and simulation data was down-sampled to the in vitro imaging frequency of 27.33Hz. * Under the condition of PP=5% with CD=5% and CD=10% no cell pair was classified as connected.

Supplementary Table ST2. Validation of classification model for predicting effective connections with different numbers of cells.

	PP=20%, CD=20%	PP=40%, CD=20%	PP=40%, CD=40%
N=43	Sensitivity=98.9%<br>Specificity=99.8%<br>Accuracy=99.6%	Sensitivity=98.9%<br>Specificity=99.7%<br>Accuracy=99.6%	Sensitivity=97.6%<br>Specificity=98.9%<br>Accuracy=98.4%
N=30	Sensitivity=99.1%<br>Specificity=99.9%<br>Accuracy=99.8%	Sensitivity=97.8%<br>Specificity=99.6%<br>Accuracy=99.3%	Sensitivity=98.7%<br>Specificity=99.4%<br>Accuracy=99.2%
N=101	Sensitivity=99.9%<br>Specificity=99.9%<br>Accuracy=99.9%	Sensitivity=97.2%<br>Specificity=99.3%<br>Accuracy=99.0%	Sensitivity=97.6%<br>Specificity=98.7%<br>Accuracy=98.1%

The classification model prediction sensitivity, specificity and accuracy for each parameter combination of propagation probability (PP) and connectivity degree (CD) were assessed on the basis of 20 simulations, each. All simulations were processed with the same algorithms as the in vitro recordings and classification performance was calculated by comparison to the ground truth given by the simulated connections. The number of cells (N) was 43 – the average of all performed in vitro recordings –, 30 – the minimum of all performed in vitro recordings – or 101 – the maximum of all performed in vitro recordings. The simulation data was down-sampled to the in vitro imaging frequency of 27.33Hz.

Supplementary Methods

Live cell fluorescence imaging

After the treatment with TTX (or controls) for two or six days, the neuronal cultures were prepared for live cell calcium imaging: After two washes with phosphate-buffered saline (PBS) to remove the TTX, cells were incubated with Fluo-4-AM solution (5 µM in imaging buffer; imaging buffer composition in mM: 144 NaCl, 2.5 KCl, 10 glucose, 10 HEPES, 2.5 $CaCl_2$, and 2.5 $MgCl_2$, pH=7.5) for 30 min. After another two washes with PBS, coverslips were placed in imaging chambers and covered with 500 µl imaging buffer.

The fluorescence signal of cells was recorded at room temperature in a setup consisting of a Nikon TI-Eclipse inverted fluorescence microscope equipped with a 10×, 0.45 NA objective. A Nikon Perfect Focus System^{TM} was used to maintain focus during perfusion. The fluorescent probes were excited by a Nikon Intensilight C-HGFI through an excitation filter with passing wavelengths of 455-485 nm. The emitted light was recorded by a -90°C water-cooled EM-CCD camera (iXon Ultra 897, Andor, Belfast, Northern Ireland) after passing an emission band-pass filter ranging from 500 to 545 nm. The dichroic longpass mirror (Semrock, Rochester, NY) had a cut-off wavelength of 495 nm.

Cells were stimulated by electric field stimulation (51 mA for 1 ms, alternating polarity) delivered through two parallel platinum electrodes spanning a field of 10 mm. Stimulation (STG 4008, Multichannel Systems) was performed in combination with a stimulus isolator (World Precision Instruments, Sarasota, FL).

Image processing and network reconstruction

Pre-processing of raw images. Before detection of the region of interest, images were filtered and cleaned of fluorescence artifacts not stemming from electrically excitable cells (i.e., neurons) by pixel-wise averaging of the fluorescence of the last five frames before electrical stimulation and the last five frames during electrical stimulation across all x- and y-coordinates. The baseline fluorescence was then subtracted from the stimulated fluorescence to obtain only responses to stimulation.

Cells were detected in the filtered images using a feature point detection algorithm as previously described [1]. Briefly, images were first filtered to correct for uneven background intensity, illumination, and camera pixel sensitivity with a box-car-average filter over a square region of 10 pixels in width around each pixel. Images were then convoluted with a Gaussian surface of revolution to compensate for camera discretization noise. Next, fluorescence intensity centers were detected by finding local maxima with an intensity of among the 1% brightest pixels in the image. Regions of interest were circular regions around the detected centers with a radius of seven pixels, corresponding to 16 µm.

The final calcium signal ΔF/F was derived from the fluorescence traces as previously described [2]. The fluorescence level for each region of interest in each time frame is given by the average intensity of all pixels assigned to that region in that time frame. The resulting fluorescence traces were corrected for the time-dependent baseline by subtracting the minimum of a moving average (width τ_1 = 0.75 s= 21 frames) during a preceding time window of τ_2 = 3 s= 82 frames. For the relative change of fluorescence compared to the time-dependent baseline, fluorescence values were divided by the baseline. An exponentially weighted moving average filter with τ_0 = 0.2 s= 6 frames half time was used as a final noise filter.

Spike estimation. Underlying action potentials were inferred from the fluorescence traces using a spike estimation algorithm [3], which deconvolutes the fluorescence trace starting from the latest time point and infers the most likely trajectory of an underlying spike train with a modified Viterbi algorithm [4]. The used trajectory template is based on the well-known calcium-dye fluorescence model by Vogelstein et al. [5], which describes a steep rise in intracellular calcium concentration upon action potential-dependent calcium influx, followed by a slow decay, and translates this calcium concentration into fluorescence intensity using a saturating static non-linearity. The most likely spike

train trajectory, underlying the given fluorescence trace, is estimated by maximizing an *a posteriori* probability distribution [6]. The spike estimation algorithm starts at the last time point and finds the most likely trajectories and their relative probabilities. Considering the next (i.e., previous to last) time step, the conditional probability is maximized, and the most likely overall trajectory is deduced after the first time point is reached.

Analysis of activity synchrony. To ensure that recorded changes in effective connectivity are not skewed by an overall change in the level of activity synchrony of the recorded cells, we calculated Cohen's kappa [7], which has been introduced as a measure of neuronal population activity synchrony in Illes et al. [8]:

$$\mathrm{K} = \frac{1}{N \cdot (N-1)} \cdot \sum_{\substack{i,j \epsilon N \\ i \neq j}} \frac{\frac{2 \cdot c_{ij} + N - s_i - s_j}{N} - \frac{s_i \cdot s_j + (N - s_i) \cdot (N - s_j)}{N^2}}{1 - \frac{s_i \cdot s_j + (N - s_i) \cdot (N - s_j)}{N^2}}$$

With N denoting the number of cells in the network, s_i and s_j denoting the number of spikes in cells i and j, and c_{ij} denoting the number of synchronous spikes in cells i and j.

Network reconstruction. We used a selection of the most common available correlation algorithms to identify statistical, causal relationships (i.e., effective network) as predictors (also called features) for the classifier. The prepared binary spike traces were analyzed with eight different algorithms:

- simple cross-correlation [9]

$$XCorr_{X \to Y} = \int_{-\infty}^{+\infty} x(t) \cdot y(t+1) dt$$

For the connection from cell X to cell Y. X(t) denotes the spikes of a cell X at time t.

- mutual information [10]

$$MI_{X \to Y} = \sum_{x=0}^{1} \sum_{y=0}^{1} p(x,y) \cdot log\left(\frac{p(x,y)}{p(x) \cdot p(y)}\right)$$

P(x) denotes the probability of a spike in cell X.

- joint entropy [11]

$$JE_{X \to Y} = -\sum_{k=1}^{3} p(cISI_k) \cdot log(p(cISI_k))$$

With cISI denoting the cross-inter spike interval – the number of time frames between a spike in cell X and the subsequent spike in cell Y.

- transfer entropy [12]

$$TE_{X \to Y} = \sum_{t=0}^{t_{max}-1} \sum_{k=1}^{3} \sum_{l=1}^{3} p(y_{t+1}, y_t^k, x_t^l) \cdot log\left(\frac{p(y_{t+1} | y_t^k, x_t^l)}{p(y_{t+1} | y_t^k)}\right)$$

With X_t^l denoting a vector whose entries are the values of X at the time steps t, t-1,…, t-k.

- generalized transfer entropy [13]

$$GTE_{X \to Y} = \sum_{t=0}^{t_{max}-1} \sum_{k=1}^{3} \sum_{l=1}^{3} p(y_{t+1}, y_t^k, x_{t+1}^l) \cdot log\left(\frac{p(y_{t+1} | y_t^k, x_{t+1}^l)}{p(y_{t+1} | y_t^k)}\right)$$

- spike count

The 'spike count' calculates the number of spikes occurring in subsequent time frames in cells X and Y.

$$SC_{X \to Y} = \sum_{t=0}^{t\_max} (x(t) | y(t+1))$$

- propagation probability
  The ‘propagation probability’ calculates the probability for a spike in cell X to be propagated and occur in cell Y in the next time frame.

$$PP_{X\rightarrow Y} = \frac{\sum_{t=0}^{t_{max}-1}(x(t)|y(t+1))}{\sum_{t=0}^{t_{max}-1}x(t)}$$

- generalized propagation probability
  The ‘generalized propagation probability’ calculates the probability for a spike in cell X to be propagated and occur in cell Y in the same or the next time frame.

$$GPP_{X\rightarrow Y} = \frac{\sum_{t=0}^{t\_max}(x(t)|y(t+1)) + \sum_{t=0}^{t\_max}(x(t)|y(t))}{\sum_{t=0}^{t\_max}x(t)}$$

The combined prediction strength of the eight used correlation measures outperformed each measure separately (Supplementary Figure S8B).

The classification model was trained and tested with the eight predictors on 60 simulated datasets each. To surely underestimate all analyzed biological data, the simulations used had a low connectivity degree (20%) and a very low propagation probability of only 5%. In the partitioning of the 120 simulations into training and test data, the prior probabilities for unconnected cells (80%) and connected cells (20 %) remained constant. We tested the performance of three different types of classifiers on our data: an adaptive boosted random forest [14], a support vector machine [15], and the naïve Bayes classifier [16] (Supplementary Figure SC). To account for the skewed prior probabilities of the two classes, we used random undersampling [14] with all three models. The classifier performing best on these data was the random forest model, with a sensitivity of 77.8%, specificity of 93.3% and accuracy of 91.1% (Supplementary Figure S8C). Therefore, this classifier was used for the network analysis of recorded cultures.

This model was validated on a wide range of initial parameters to ensure correct classifications on the analyzed in vitro data: 20 simulations with connectivity degrees of 5%, 10%, 20%, 40%, 60% and 80%, each in combination with a propagation probability of 5%, 10%, 20%, 40%, 60% and 80% (Supplementary Table ST1). All these 720 simulations were set up with a cell number of 43 – the average of our in vitro recordings. As the classification model classifies each possible connection separately, the number of cells per recordings or simulation had no effect on the sensitivity, specificity or accuracy of the classification model: Simulations with initial parameters similar to the in vitro data were set up with 43 (average of in vitro data), 30 (minimum of in vitro data) and 101 (maximum of in vitro data) cells. The performance of the classification model only slightly varied (Supplementary Table ST2), which may most likely be due to the greater sample size with more cells.

Network analysis. From a multitude of possible network topology properties [17], we chose to analyze 12 different features that can be transferred to an underlying biological rationale. Detailed mathematical descriptions of these network analysis parameters can be found in the Brain Connectivity Toolbox documentation by Rubinov et al. (RRID: SCR_004841) [17].

Degree properties

- In-degree: Number of inward links connected to a cell.

$$k_i^{in} = \sum_{j\in N} a_{ji}$$

  With i and j denoting the indices of all N cells in the network and a_{ij} denoting the connection status from cell i to cell j (a_{ij}=1 if connected and a_{ij}=0 if not connected).
- Out-degree: Number of outward links connected to a cell.

$$k_i^{out} = \sum_{j\in N} a_{ij}$$

- Out-per-in-degree: Ratio of outward-to-inward links connected to a cell.

$$o_i = \frac{k_i^{out}}{k_i^{in}}$$

Connection properties

- Propagation probability: Probability of a spike to be propagated across an existing connection. A spike in a target cell is defined as propagated if it occurs in the same or the following time frame as the spike in the source cell

$$w_{ij} = \frac{\sum_{t=0}^{t_{max}-1}\big(x(t)\big|y(t+1)\big) + \sum_{t=0}^{t_{max}-1}\big(x(t)\big|y(t)\big)}{\sum_{t=0}^{t_{max}-1} x(t)}$$

- Distance: Physical distance (in µm) between the cells.
- Connectivity degree: Percentage of the total number of possible connections that exist in the network.

$$c = \frac{\sum_{i,j\in N} a_{ij}}{N \cdot (N-1)}$$

Path length properties

- Characteristic path length of binary network: Average length of the shortest path between two random cells in the network.

$$L = \frac{1}{N} \cdot \sum_{i\epsilon N} \frac{\sum_{j\epsilon N, j\neq i} d_{ij}}{N-1}$$

With d_{ij} denoting the distance between nodes i and j.

- Characteristic path length of weighted network: Average length of the shortest path between two random cells in the network, weighted by each intermediate connection’s strength.

$$L^w = \frac{1}{N} \cdot \sum_{i\epsilon N} \frac{\sum_{j\epsilon N, j\neq i} d_{ij}^w}{N-1}$$

With d_{ij}^w denoting the weighted distance between nodes i and j.

- Global efficiency: The efficiency with which a signal can travel from one cell to another in the network (i.e., number of stopovers). Inversely related to characteristic path length.

$$E^w = \frac{1}{N} \cdot \sum_{i\epsilon N} \frac{\sum_{j\epsilon N, j\neq i} \frac{1}{d_{ij}^w}}{N-1}$$

Clustering properties

- Clustering coefficient: Fraction of connection partners of a cell, which are connected with each other.

$$C = \frac{1}{N} \cdot \sum_{i\epsilon N} \frac{\frac{1}{2} \cdot \sum_{j,h\epsilon N} (a_{ij} + a_{ji})(a_{ih} + a_{hi})(a_{jh} + a_{hj})}{\left(k_i^{out} + k_i^{in}\right) \cdot \left(k_i^{out} + k_i^{in} - 1\right) - 2\sum_{j\epsilon N} a_{ij} \cdot a_{ji}}$$

- Modularity degree: The extent to which a network can be subdivided into modules, which are groups of nodes that are highly connected within the module and have a minimal number of connections to other modules.

$$Q = \frac{1}{l} \cdot \sum_{i,j\epsilon N} \left(a_{ij} - \frac{k_{ij}^{out} \cdot k_{ij}^{in}}{l}\right) \cdot \delta(m_i, m_j)$$

With l denoting the number of links in the network and m_i denoting the module that contains node i.

Structure properties

- Betweenness centrality: The fraction of all shortest paths in the network that go through a cell. Cells with high values of betweenness centrality participate in a large number of shortest paths and thus play a particularly important role in the signal processing cascade.

$$b_i = \frac{1}{(N-1)\cdot(N-2)} \cdot \sum_{\substack{h,j\varepsilon N \\ h\neq i, h\neq j, j\neq i}} \frac{\rho_{hj}(i)}{\rho_{hj}}$$

  With $\rho_{hj}(i)$ denoting the number of shortest paths between nodes h and j that pass through i.

- Assortativity coefficient: The extent to which cells show preferential attachment. An assortativity coefficient near zero corresponds to random connectivity. Positive values arise from connections between cells of similar degrees, whereas negative values (which is typical in neuronal cultures [18]) arise from connections between cells of different degrees.

$$r^w = \frac{\frac{1}{l}\cdot\sum_{i,j\epsilon L} w_{ij}k_i^w k_j^w - \left(\frac{1}{l}\cdot\sum_{i,j\epsilon L}\frac{1}{2}w_{ij}\left(k_i^w + k_j^w\right)\right)^2}{\frac{1}{l}\cdot\sum_{i,j\epsilon L}\frac{1}{2}w_{ij}\left(k_i^{w2} + k_j^{w2}\right) - \left(\frac{1}{l}\cdot\sum_{i,j\epsilon L}\frac{1}{2}w_{ij}\left(k_i^w + k_j^w\right)\right)^2}$$

Simulations

Simulations were generated with a leaky integrate-and-fire neuronal model [19] with NEST [20] as binary spike time data with 1-ms time resolution and random connection topology. The number of cells per simulation was either 43 (the average of all our in vitro recordings), 30 (the minimum in our in vitro recordings) or 101 (the maximum in our in vitro recordings) and the simulated duration was 18.5 minutes – similar to our in vitro recordings. Other initial parameters of the simulations are given in the manuscript text, tables and figures with the different used datasets.

The simulated binary spike time traces were converted into fluorescence data with the common model by Vogelstein et al. [5,13] and then down-sampled to the low time resolution of our in vitro recording frame rate of 27.33 Hz by discarding intermediate time steps. In addition to the Gaussian noise introduced in the fluorescence model, we added white noise to compensate for fluctuations in imaging (e.g., dark current, camera intensity fluctuations and focus vibrations). To ensure comparability of the simulated in silico and the recorded in vitro data, the resulting fluorescence traces were filtered and analyzed with the same scripts and parameters as the recordings. All classification model validations were conducted on simulated data that was processed and down-sampled to 27.33 Hz as described.

The codes for these simulations and all analysis scripts are available in the GitHub repository (www.github.com/janawrosch/effective_connectivity).

References

1 Sbalzarini, I. F. & Koumoutsakos, P. Feature point tracking and trajectory analysis for video imaging in cell biology. *Journal of structural biology* **151**, 182-195, doi:10.1016/j.jsb.2005.06.002 (2005).

2 Jia, H., Rochefort, N. L., Chen, X. & Konnerth, A. In vivo two-photon imaging of sensory-evoked dendritic calcium signals in cortical neurons. *Nature protocols* **6**, 28-35, doi:10.1038/nprot.2010.169 (2011).

3 Deneux, T. *et al.* Accurate spike estimation from noisy calcium signals for ultrafast three-dimensional imaging of large neuronal populations in vivo. *Nature communications* **7**, 12190, doi:10.1038/ncomms12190 (2016).

4 Viterbi, A. Error bounds for convolutional codes and an asymptotically optimum decoding algorithm. *IEEE Transactions on Information Theory* **13**, 260-269, doi:10.1109/TIT.1967.1054010 (1967).

5 Vogelstein, J. T. *et al.* Spike inference from calcium imaging using sequential Monte Carlo methods. *Biophysical journal* **97**, 636-655, doi:10.1016/j.bpj.2008.08.005 (2009).

6 Pnevmatikakis, E. A. *et al.* Fast spatiotemporal smoothing of calcium measurements in dendritic trees. *PLoS computational biology* **8**, e1002569, doi:10.1371/journal.pcbi.1002569 (2012).

7 Cohen, J. A Coefficient of Agreement for Nominal Scales. *Educational and Psychological Measurement* **20**, 37-46, doi:doi:10.1177/001316446002000104 (1960).

8 Illes, S., Theiss, S., Hartung, H. P., Siebler, M. & Dihne, M. Niche-dependent development of functional neuronal networks from embryonic stem cell-derived neural populations. *BMC neuroscience* **10**, 93, doi:10.1186/1471-2202-10-93 (2009).

9 Salinas, E. & Sejnowski, T. J. Correlated neuronal activity and the flow of neural information. *Nature reviews. Neuroscience* **2**, 539-550, doi:10.1038/35086012 (2001).

10 Xu, J., Liu, Z.-r., Liu, R. & Yang, Q.-f. Information transmission in human cerebral cortex. *Physica D: Nonlinear Phenomena* **106**, 363-374 (1997).

11 Lungarella, M., Pitti, A. & Kuniyoshi, Y. Information transfer at multiple scales. *Physical Review E* **76**, 056117 (2007).

12 Schreiber, T. Measuring information transfer. *Physical review letters* **85**, 461-464, doi:10.1103/PhysRevLett.85.461 (2000).

13 Stetter, O., Battaglia, D., Soriano, J. & Geisel, T. Model-free reconstruction of excitatory neuronal connectivity from calcium imaging signals. *PLoS computational biology* **8**, e1002653, doi:10.1371/journal.pcbi.1002653 (2012).

14 Seiffert, C., Khoshgoftaar, T. M., Van Hulse, J. & Napolitano, A. RUSBoost: A hybrid approach to alleviating class imbalance. *IEEE Transactions on Systems, Man, and Cybernetics-Part A: Systems and Humans* **40**, 185-197 (2010).

15 Cortes, C. & Vapnik, V. Support-vector networks. *Machine Learning* **20**, 273-297, doi:10.1007/bf00994018 (1995).

16 Mumford, J. A. & Ramsey, J. D. Bayesian networks for fMRI: A primer. *NeuroImage* **86**, 573-582, doi:10.1016/j.neuroimage.2013.10.020 (2014).

17 Rubinov, M. & Sporns, O. Complex network measures of brain connectivity: uses and interpretations. *NeuroImage* **52**, 1059-1069, doi:10.1016/j.neuroimage.2009.10.003 (2010).

18 Barabási, A.-L. (Basic Books, 2002).

19 Rotter, S. & Diesmann, M. Exact digital simulation of time-invariant linear systems with applications to neuronal modeling. *Biological cybernetics* **81**, 381-402 (1999).

20 Bos, H. M., Abigail; Peyser, Alexander; Hahne, Jan; Helias, Moritz; Kunkel, Susanne; Ippen, Tammo; Eppler, Jochen Martin; Schmidt, Maximilian; Seeholzer, Alex; Djurfeldt, Mikael; Diaz, Sandra; Morén, Janne; Deepu, Rajalekshmi; Stocco, Teo; Deger, Moritz; Michler, Frank; Plesser, Hans Ekkehard. NEST 2.10.0. doi:10.5281/zenodo.44222 (2015).
